# Pepsin Egg White Hydrolysate Ameliorates Obesity-Related Oxidative Stress, Inflammation and Steatosis in Zucker Fatty Rats

**DOI:** 10.1371/journal.pone.0151193

**Published:** 2016-03-17

**Authors:** M. Garcés-Rimón, C. González, J. A. Uranga, V. López-Miranda, R. López-Fandiño, M. Miguel

**Affiliations:** 1 Instituto de Investigación en Ciencias de Alimentación (CIAL, CSIC-UAM), Madrid, Spain; 2 Departamento de Ciencias Básicas de la Salud, Facultad de Ciencias de la Salud, Universidad Rey Juan Carlos, Alcorcón, Madrid, Spain; East Tennessee State University, UNITED STATES

## Abstract

The aim of this work was to evaluate the effect of the administration of egg white hydrolysates on obesity-related disorders, with a focus on lipid metabolism, inflammation and oxidative stress, in Zucker fatty rats. Obese Zucker rats received water, pepsin egg white hydrolysate (750 mg/kg/day) or *Rhizopus* aminopeptidase egg white hydrolysate (750 mg/kg/day) for 12 weeks. Lean Zucker rats received water. Body weight, solid and liquid intakes were weekly measured. At the end of the study, urine, faeces, different organs and blood samples were collected. The consumption of egg white hydrolysed with pepsin significantly decreased the epididymal adipose tissue, improved hepatic steatosis, and lowered plasmatic concentration of free fatty acids in the obese animals. It also decreased plasma levels of tumor necrosis factor-alpha and reduced oxidative stress. Pepsin egg white hydrolysate could be used as a tool to improve obesity-related complications.

## Introduction

Obesity is one of the most common and important health concerns that our society faces up, whose prevalence has led the World Health Organization to define it as a global epidemic. Obesity, in particular central adiposity, increases the levels of reactive oxygen species (ROS) that promote the expression and secretion of inflammatory adipokines [1–3]. Inflammation and oxidative stress in the adipose tissue have been recognized as key components in the development of metabolic syndrome [4]. Moreover, visceral adiposity leads to accumulation of lipids in the liver due to increased fatty acid synthesis and inhibited fatty acid utilization. This situation, linked to a chronic inflammation state and oxidative stress, contributes to the progression of steatosis or fatty liver, which could result in cirrhosis or hepatocellular carcinoma [5].

Lifestyle modifications including diet, weight loss and exercise are the most appropriate initial therapeutic interventions for obese patients. When pharmacologic and surgical options are considered, there are several risks associated, which point at dietary measures as the safest and most cost-effective alternatives. In this respect, bioactive peptides derived from food proteins have shown a promising future for the management of complex human health conditions due to their potential pleiotropic effects [6].

The benefits of using food protein hydrolysates with a multiple-targeting purpose can be illustrated by the studies of different researchers, who showed that the peptides contained in a hydrolysate of egg white with pepsin reduce hypertension, oxidative stress and hyperlipidemia in spontaneously hypertensive rats (SHR) [7–10]. Similarly, a hydrolysate of lysozyme with alcalase effectively improves glycaemia and limits renovascular damage in diabetic Zucker rats [11]. In addition, the peptides contained in a novel fermented milk whey product show positive effects on *in vivo* models of dyslipidemia, insulin resistance and hypertension [12].

In an attempt to select egg-derived bioactive peptides with potential to treat cardiometabolic disorders, such as obesity, dyslipidemia, diabetes and hypertension, we carried out an *in vitro* screening of egg white hydrolysates produced with food-grade enzymes from different sources, on the basis of their antioxidant, anti-inflammatory, bile acid binding (hypocholesterolemic related marker), dipeptidyl peptidase IV (DPP IV) (glucose metabolism related marker) and angiotensin converting enzyme (ACE)-inhibitory activities [13]. The hydrolysate of egg white with pepsin presents a high ACE-inhibitory activity, as well as important peroxy radical-trapping and bile acid-binding activities, while the hydrolysate with aminopeptidase from *Rhizopus oryzae* shows an important peroxy radical-trapping activity and potential hypocholesterolemic activity. Furthermore, both hydrolysates prevent oxidative damage in the macrophage RAW 264.7 cell line and exhibit a moderate inhibitory activity towards the enzyme DDP IV, and thus, they could be able to prevent the degradation of the incretin hormones that stimulate glucose-dependent insulin secretion [13].

The multiple properties exerted by the peptides released by these enzymes suggest that they could target several symptoms of a complex disease, such as metabolic syndrome. In this work we address the beneficial effects of the dietary supplementation with the hydrolysates of egg white with pepsin and *Rhizopus* aminopeptidase on Zucker fatty rats, an experimental model of obesity, with emphasis on lipid metabolism, inflammation and oxidative stress.

## Materials and Methods

### General protocol in rats

Thirty male 8 week-old Zucker fatty rats, weighing 250–275 g, and ten 8 week-old male Zucker lean rats, weighing 150–175 g, all purchased from Charles River Laboratories (Charles River Laboratories, Barcelona, Spain), were used in this study. During the experimental period, the animals were maintained at temperature (23°C) with 12 h light/dark cycles, and they were fed *ad libitum* with a solid standard diet (A04 Panlab, Barcelona, Spain). The obese Zucker rats were randomly divided into three groups of ten animals. The obese animals received the following drinking fluids: tap water (control), egg white hydrolysed with pepsin or egg white hydrolysed with aminopeptidase, both dissolved in tap water to give 750 mg/kg/day of these products to the animals for 12 weeks. The lean Zucker rats were in turn fed the standard diet and tap water until 20^th^ week of life. This group of animals was used as control for normal values of this rat strain.

To prepare the hydrolysates, commercial pasteurized egg white was hydrolysed with BC Pepsin 1:3000 (E.C. 3.4.23.1; from pork stomach, E:S: 2:100 w:w, pH 2.0, 38°C), and Peptidase 433P (E.C. 3.4.11.1; from *Rhizopus oryzae*, E:S: 2:100 w:w, pH 7.0, 50°C), purchased from Biocatalysts (Cardiff, United Kingdom), for 8 and 24 h respectively. Enzyme inactivation was achieved by increasing the pH to 7.0 with 5N NaOH, in the case of pepsin, and heating at 95°C for 15 min, in the case of the aminopeptidase. The hydrolysates were centrifuged at 2500 x g for 15 min and the supernatants were frozen at -20°C and lyophilised. The daily doses of 750 mg/kg were selected according to the results obtained after *in vitro* studies [13], and from previous *in vivo* studies using egg white hydrolysates in SHR [10, 14, 15].

The body weight of the animals was recorded weekly up to the 20^th^ week of life. Daily intake of drinking fluids and of freely accessible feed was also estimated weekly in the animals from the different groups throughout the experimental period.

During the last week of treatment, the animals were placed in metabolic cages and urine and faeces were collected during 16 h. Faeces were weighted and their fat content determined by the Soxhlet method [16]. Fat Apparent Digestibility (FAD) was calculated as percentage of the fat intake, estimated by taking into account the composition of diets.

At the end of the experimental period (20^th^ week of life), after an over-night fasting, the rats were deeply anaesthetized with an intraperitoneal injection of equitesin (2.1 g chloral hydrate; 1.06 g MgSO4; 0.46 g pentobarbital; 21.4 ml propylene glycol and 5.7 ml ethanol in H2O) at a dosage of 0.3 ml/kg body weight and sacrificed by decapitation. Blood was obtained to carry out the following biochemical determinations in plasma: total cholesterol, triglycerides, free fatty acids (FFAs), malonyldialdehyde (MDA), antioxidant capacity, TNF-αand adiponectin. Epididymal fat and liver were immediately excised and weighed, and the percentage of wet organ-weight to body weight ratio was calculated for each organ. Histopathological analyses of both tissues were performed and reduced glutathione in the liver was also evaluated.

The experiments were designed and performed in accordance with the European and Spanish legislation on care and use of experimental animals (2010/63/UE; Real Decreto 53/2013), and were approved by the Ethics Committee of the Universidad Rey Juan Carlos. The sacrifice was performed under equitesin anesthesia, and all efforts were made to minimize suffering. The physical condition of the animals was observed every day and monitored once a week during the experimental period. All the animals survived in perfect conditions during the study.

#### Plasma and tissue preparations

Blood samples were collected into tubes containing lithium heparin as anticoagulant. These samples were centrifuged at 2500 g for 20 min at 4°C to obtain the plasma, which was divided into aliquots and kept frozen at -80°C until analysis. Liver samples were homogenized at 4°C in a Potter with PBS (0.01 M PBS, 0.15 M NaCl, pH 7.4), centrifuged at 5000 g for 15 min at 4°C and the supernatants were recovered and kept frozen at -80°C until used for the evaluation of reduced glutathione. The protein content of the homogenates was determined by the Bio-Rad protein assay (Bio-Rad Laboratories, Hercules, CA, USA), using bovine serum albumin as standard.

#### Triglycerides and total cholesterol

The lipid profile (triglycerides and total cholesterol) was assayed using enzymatic and colorimetric methods with commercial kits (MI41031 and MI41021; Spinreact S.A/S.A.U, Spain). The concentrations were determined at 450 nm with a spectrophotometer (Biotek HT Sinergy, USA).

#### Plasma free fatty acids

The concentration of plasma free-fatty acids (FFAs) was determined using an EnzyChrom Free Fatty Acid Assay Kit, (F6180-50; BioAssay Systems, United States). The optical density was measured at 570 nm as above. Different concentrations of palmitic acid were used as standards.

#### Plasma malondialdehyde

Levels of plasma malondialdehyde (MDA) were measured by the thiobarbituric acid (TBA) assay previously described by Manso et al. [10]. Briefly, plasma samples were incubated with NaOH for 30 min and mixed with 20% trichloroacetic acid in 0.6 M HCl (1:1, v/v). The tubes were kept on ice for 20 min to precipitate interfering components. Samples were then centrifuged at 1500 g for 15 min before adding TBA (Sigma-Aldrich, 120 mM in 260 mM Tris, pH 7) to the supernatants in a proportion of 1:5 (v/v) and the mixture was heated at 97°C for 30 min. The optical density was measured at 535 nm at 20°C.

#### Plasma antioxidant capacity

The antioxidant capacity was measured by the oxygen radical absorbance capacity (ORAC) assay previously described [10]. ORAC values were quantified by a fluorimeter Polarstar Galaxy plate reader (BMG Labtechnologies GmbH, Germany) and expressed as μmol of Trolox (Sigma) equivalent/μl of plasma.

#### Plasma adiponectin and TNF-α

Plasma adiponectin concentration was determined using a rat adiponectin ELISA kit (KRP0041; Invitrogen, Life Technologies S.A., Spain), and plasma TNF-α concentration was determined using a rat TNF-α ELISA kit (ER3TNFA; Invitrogen) according to the manufacturer’s instructions.

#### Histopathological analysis

Livers and white and brown adipose tissues were obtained at the end of the experimental period from the 10 animals of each experimental group. Samples were fixed in buffered 10% formalin and embedded in paraffin. Tissues were cut in sections of 5 μm and stained with hematoxylin-eosin (HE) for general analysis and with Van Giesson stain to reveal liver fibrosis. They were studied under a Zeiss Axioskop 2 microscope (Carl Zeiss Microscopy, LLC, United States) equipped with the image analysis software package AxioVision 4.6. A qualitative analysis was made in 2 to 4 slices of liver and adipose tissue per animal. Besides, adipocyte size was indirectly measured counting the number of cells per field under a 20x objective, in the case of the white adipocytes, and under 40x in the brown fat samples.

#### Liver glutathione determination

Reduced glutathione levels were determined by the monochlorobimane fluorimetric method [17]. The final assay mixture (100 μl) contained 90 μl of the liver samples, or different concentrations (0.001–10 mM) of reduced glutathione (Sigma-Aldrich Chemie CMBh, Germany) in PBS (pH 7.4), as a standard, and 10 μl of glutathione S-transfer (1 U/ml) from equine liver and 1 mM monochlorobimane (Fluka Biochemical, Switzerland). The samples were incubated in the dark at room temperature for 30 min. The reduced glutathione adduct was measured using a fluorimeter (BMG Labtechnologies GmbH, Germany), with excitation at 380 nm and emission at 470 nm.

#### Statistical analysis

The results are expressed as mean values ± SEM. for a minimum of 8 rats, and were analyzed by the Student t test and one or two-way analysis of variance (ANOVA), using the GraphPad Prism 4 software (San Diego, CA). Differences between the groups were assessed by the Bonferroni test. Differences between the means were considered to be significant when P< 0.05.

## Results

### Food and drink intakes and body and organ weights

**Fig 1:**
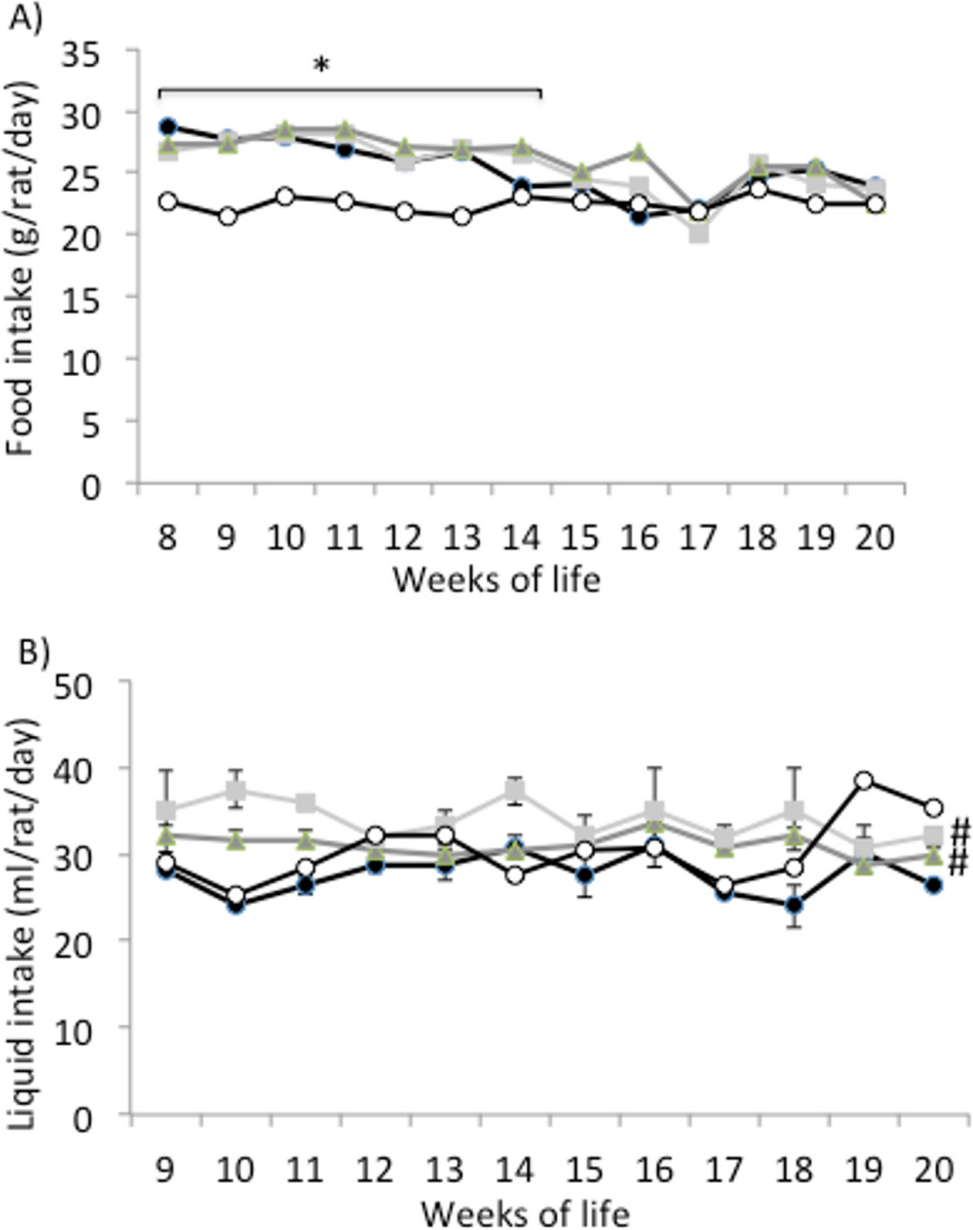
Food and liquid intake. A) Food intake (g/rat/day) and B) liquid intake (g/rat/day) of lean Zucker rats (white), obese Zucker rats that received water (black), obese Zucker rats that received 750 mg/kg/day of pepsin egg white hydrolysate (light grey) and obese Zucker rats that received 750 mg/kg/day of aminopeptidase egg white hydrolysate (dark grey) for 12 weeks. Values are means ± SEM (n = 10). * P<0.05 obese vs lean rats, #P<0.05 obese rats treated with egg white hydrolysates vs control obese rats.

Food intake was significantly higher in the obese than in the lean Zucker rats during the first 6 weeks of the study (Fig 1A). From week 14^th^ onwards, food intake was similar in the obese and lean animals. No differences in food intake were detected between the obese animals taking water and those taking the egg white hydrolysates. On the other hand, there were no differences in drink intake between the lean animals and the obese animals taking water, although the obese rats taking the hydrolysates drunk more liquid than the obese controls (Fig 1B).

The volume of urine excreted in 16 hours at the end of the study was significantly higher in the obese control rats than in the lean rats. Despite the differences mentioned in liquid intake, no significant differences were observed among the obese animals on urine excretion (Table 1). The amount of feces excreted in 16 hours was also higher in the obese rats as compared with the lean rats, as it was the FAD. These parameters were not affected by the administration of the egg white hydrolysates either (Table 1).

**Table 1:** Faeces, fat apparently digested (FAD) and urine measured during 16 h in the last week of treatment of lean Zucker rats, obese Zucker rats that received water (control) and obese Zucker rats that received 750 mg/kg/day of pepsin and aminopeptidase egg white hydrolysates for 12 weeks. Values are means ± SEM (n = 10).

Experimental groups	Lean Zucker	Control obese Zucker	Obese Zucker treated with pepsin hydrolysate	Obese Zucker treated with aminopeptidase hydrolysate
**Faeces (g)**	4.38±0.39	7.01±0.40*	7.21±0.67	7.16±0.56
**% FAD**	49.8±15.67	86.26±5.67*	78.51±12.72	85.95±1.51
**Urine (ml)**	3.3±0.48	5.42±0.72*	7.02±0.70	7.13±0.90

* P<0.05 obese vs lean rats.

**Fig 2:**
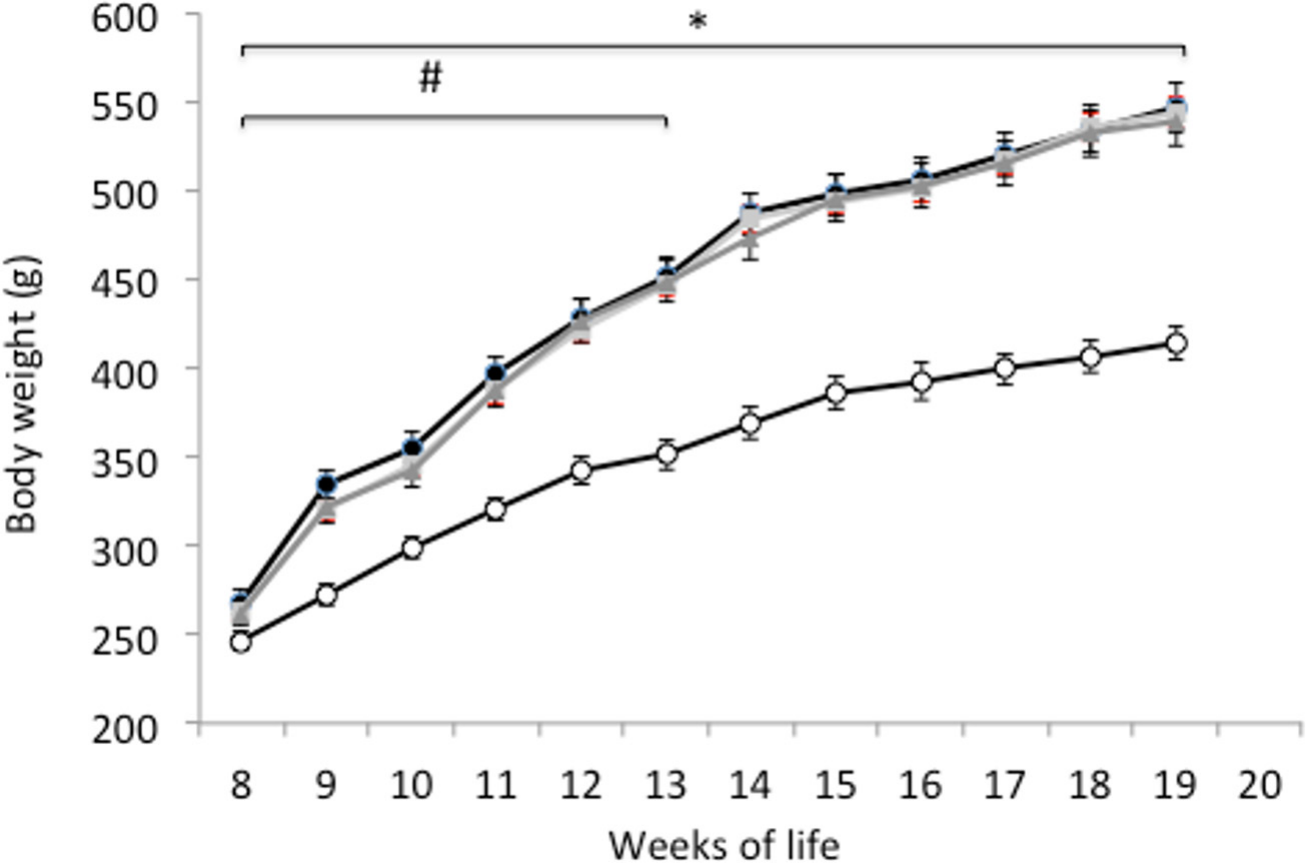
Body weight. Body weight (g) of lean Zucker rats (white), obese Zucker rats that received water (black), obese Zucker rats that received 750 mg/kg/day of pepsin egg white hydrolysate (light grey) and obese Zucker rats that received 750 mg/kg/day of aminopeptidase egg white hydrolysate (dark grey) for 12 weeks. Values are means ± SEM (n = 10). * P<0.05 obese vs lean rats.

As expected, the body weight of the lean Zucker rats was the lowest throughout the whole study (Fig 2). The obese animals taking the egg white hydrolysates gained significantly less weight than the obese controls during the first 4 weeks, but there were no differences in weight among the three obese animal groups at the end of the study (Fig 2). Absolute and relative weights of the epididymal adipose tissue were higher in the control obese animals as compared with the lean Zucker rats, although the administration of egg white hydrolysed with pepsin significantly reduced these values (Table 2). In the obese control group, the absolute liver weight almost doubled that of the lean animals and the relative liver weight was also higher. The obese animals, which consumed egg white hydrolysed with pepsin, showed a slightly decreased absolute and relative liver weight, but no significant differences were found. The relative kidney weight was significantly lower in the control obese Zucker rats than in the lean rats (Table 2).

**Table 2:** Body and organs weight at the end of the study period of lean Zucker rats, obese Zucker rats that received water (control), and obese Zucker rats that received 750 mg/kg/day of pepsin and aminopeptidase egg white hydrolysates for 12 weeks. Values are means ± SEM (n = 10).

Experimental groups	Lean Zucker	Control obese Zucker	Obese Zucker treated with pepsin hydrolysate	Obese Zucker treated with aminopeptidase hydrolysate
**Body weight**	414.0±9.10	546.60±14.04*	544.0±8.17	538.10±12.84
**Liver weight (g)**	14.87±0.73	28.64±0.95*	26.06±1.02	27.40±1.15
**Liver weight (g/100g body weight)**	3.7±0.13	5.4±0.19*	4.9±0.19	5.3±0.17
**Kidney weight (g)**	2.89±0.18	2.84±0.21	2.59±0.05	2.82±0.20
**Kidney weight (g/100g body weight)**	0.72±0.04	0.053±0.03*	0.48±0.01	0.54±0.03
**Epididymal adipose tissue (g)**	6.37±0.31	14.80±0.24*	13.41±0.2^♯^	14.18±0.44
**Epididymal adipose tissue (g/100g body weight)**	1.60±0.006	2.72±0.07*	2.47±0.02^♯^	2.64±0.09

* P<0.05 obese vs lean rats

#P<0.05 obese rats treated with egg white hydrolysates vs control obese rats.

### Histology of adipose and liver tissues

**Fig 3:**
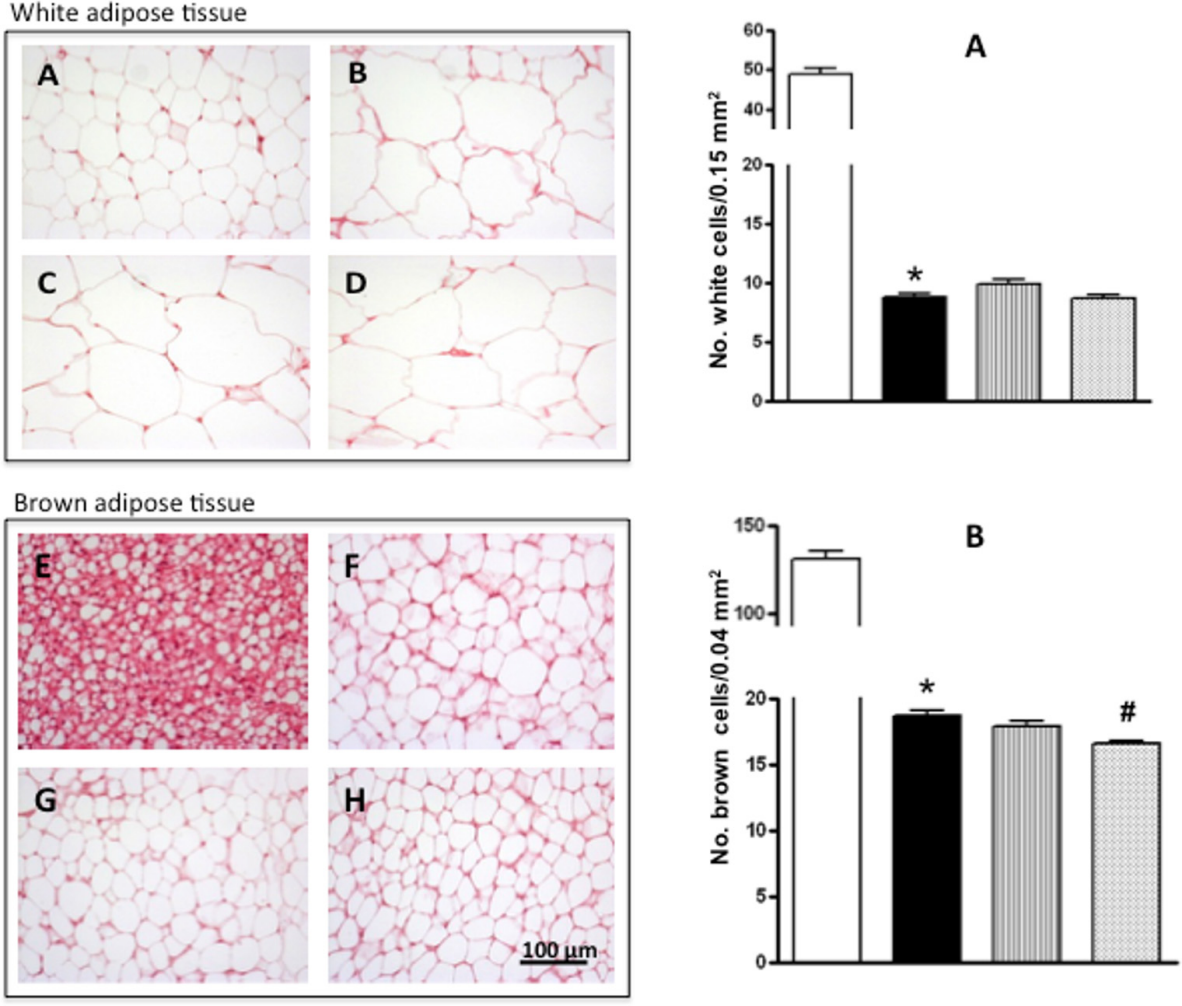
Histology of white and brown adipose tissue. *Left*: Adipocytes stained with hematoxylin-eoxin from white adipose tissue (20x, A-D) and from brown adipose tissue (40x, E-H) of lean Zucker rats (A, E) and obese Zucker rats that received water (B, F), obese Zucker rats that received 750 mg/kg/day of pepsin egg white hydrolysate (C, G) and obese Zucker rats that received 750 mg/kg/day of aminopeptidase egg white hydrolysate (D, H) for 12 weeks. *Right*: Number of adipocytes in the white adipose tissue (top graph) and brown adipose tissue (bottom graph) of lean Zucker rats (white), obese Zucker rats that received water (black), obese Zucker rats that received 750 mg/kg/day of pepsin egg white hydrolysate (light grey) and obese Zucker rats that received 750 mg/kg/day of aminopeptidase egg white hydrolysate (dark grey), for 12 weeks. Values are means ± SEM (n = 10). * P<0.01 obese vs lean rats, #P<0.01 obese rats treated with pepsin egg white hydrolysate vs control obese rats.

The histological studies of the epididymal white adipose tissue and interscapular brown adipose tissue (Fig 3) indicated that the size of the epididymal and interscapular adipocytes was greater in the obese than in the lean rats (there were 5 and 8 times less adipocytes per unit area, respectively). There was no effect of either of the egg white hydrolysates on the average size of the white adipose tissue adipocytes.

**Fig 4:**
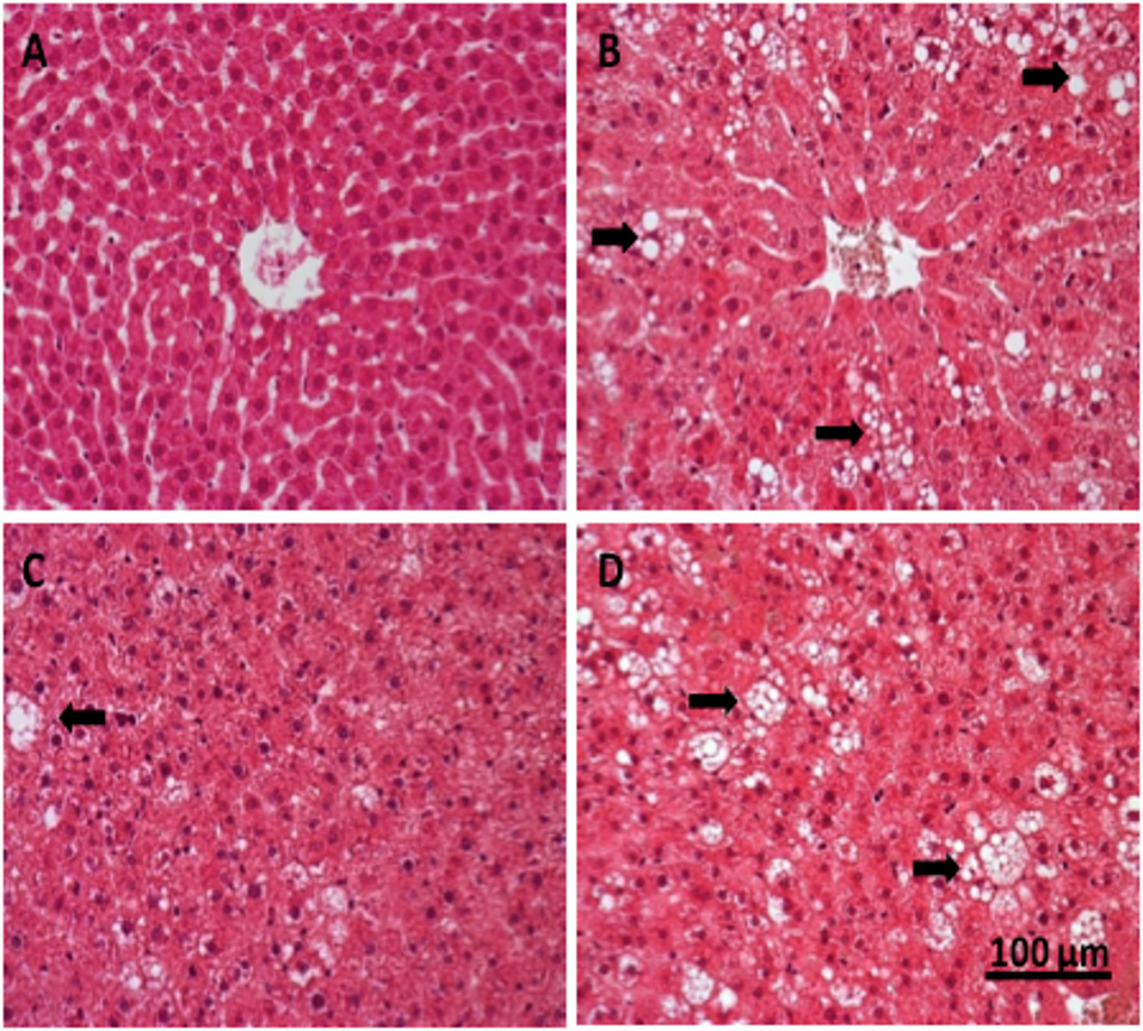
Histology of liver tissue. Liver tissue stained with hematoxylin-eoxin (40x) of lean Zucker rats (A), obese Zucker rats that received water (B), obese Zucker rats that received 750 mg/kg/day of pepsin egg white hydrolysate (C) and obese Zucker rats that received 750 mg/kg/day of aminopeptidase egg white hydrolysate (D) for 12 weeks. Liposomes are indicated by arrows.

The histological study of liver tissues stained with haematoxylin and eosin (Fig 4) revealed clear differences among the experimental groups. The liver of lean Zucker rats did not show any sign of pathology, nor extravascular T of B lymphocytes (Fig 4A), while that of obese Zucker rats that took water presented a marked steatosis, with heterogeneous distribution of fat microvesicules and mild lymphocyte infiltration (Fig 4B). However, the obese animals that received the hydrolysate of egg white with pepsin showed a strikingly reduced liver steatosis, with less and smaller fat vesicles (Fig 4C). This improvement of the liver condition was not observed in the group treated with the hydrolysate produced with aminopeptidase (Fig 4D). In view of the positive results obtained with the hydrolysate of egg white with pepsin, the effect of this product on lipid metabolism and oxidative stress in the obese Zucker rats was subsequently examined.

### Lipid metabolism

Table 3 shows the plasma concentrations of total cholesterol, triglycerides, and FFAs, which were significantly greater in the obese Zucker rats that took water as compared with the lean rats. The administration of egg white hydrolysed with pepsin to the obese animals led to significantly lower levels of FFAs and to slightly lower, albeit not significant, concentrations of total cholesterol and triglycerides.

**Table 3:** Lipid biomarkers in plasma of lean Zucker rats, obese Zucker rats that received water (control), and obese Zucker rats that received 750 mg/kg/day of pepsin for 12 weeks. Values are means ± SEM (n = 10).

Experimental groups	Lean Zucker	Control obese Zucker	Obese Zucker treated with pepsin hydrolysate
**Cholesterol (mg/dl)**	157.2±8.6	218.5±5.3 *	206.2±7.8
**Triglycerides (mg/dl)**	171.5±5.0	328.5±19.1 *	306.6±8.7
**FFA (mM)**	0.31±0.015	0.37±0.014 *	0.33±0.01 ^♯^

* P<0.05 obese vs lean rats

#P<0.05 obese rats treated with egg white hydrolysate vs control obese rats.

### Inflammation and oxidative stress markers

**Fig 5:**
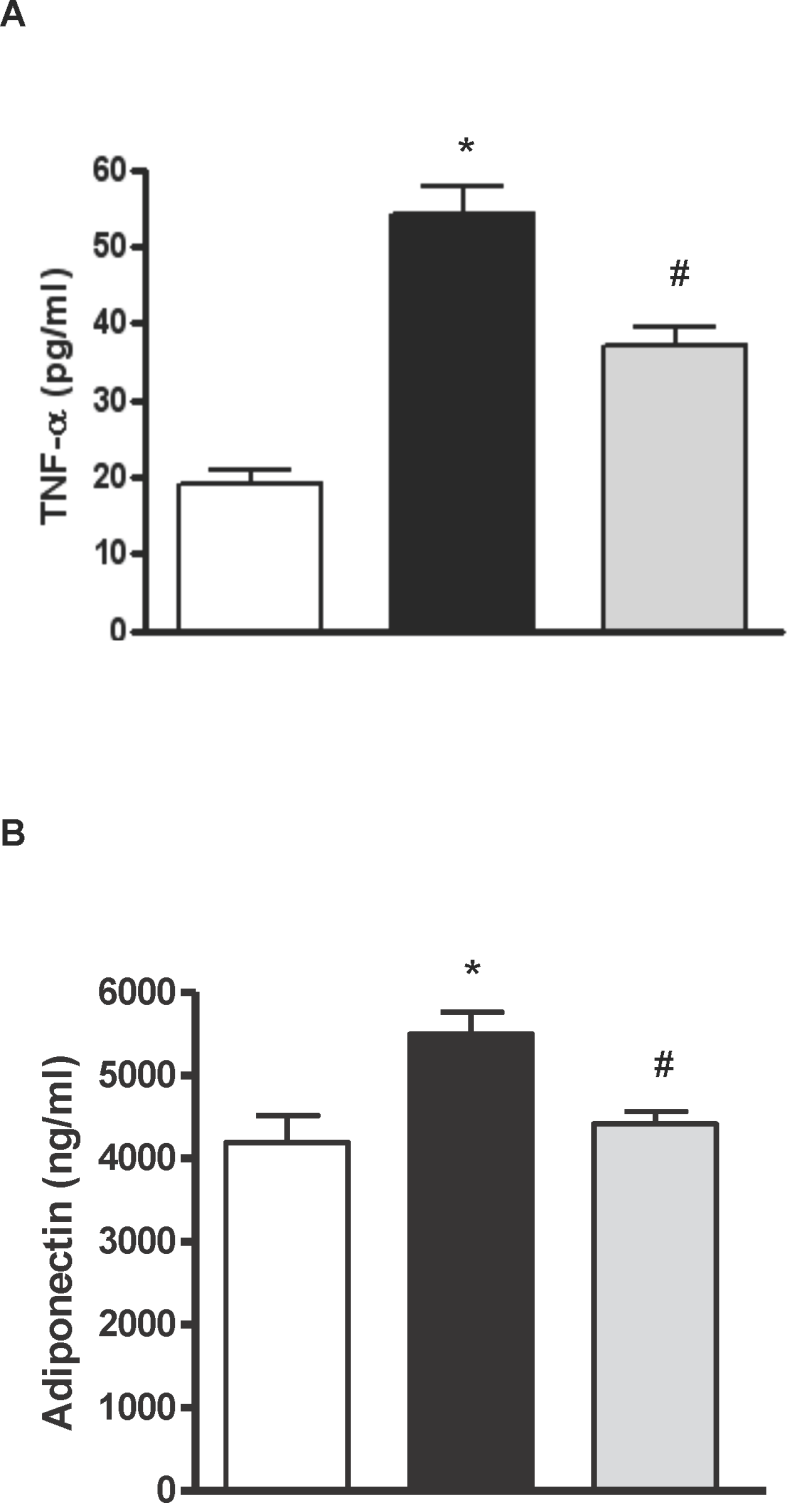
TNF-α and adiponectin. Histograms of A) tumor necrosis factor alpha (TNF-α) (pg/ml) and B) adiponectin (ng/ml) in plasma of lean Zucker rats (white), obese Zucker rats that received water (black) and obese Zucker rats that received 750 mg/kg/day of pepsin egg white hydrolysate (light grey) for 12 weeks. Values are means ± SEM (n = 10). * P<0.05 obese vs lean rats, #P<0.05 obese rats treated with pepsin egg white hydrolysate vs control obese rats.

**Fig 6:**
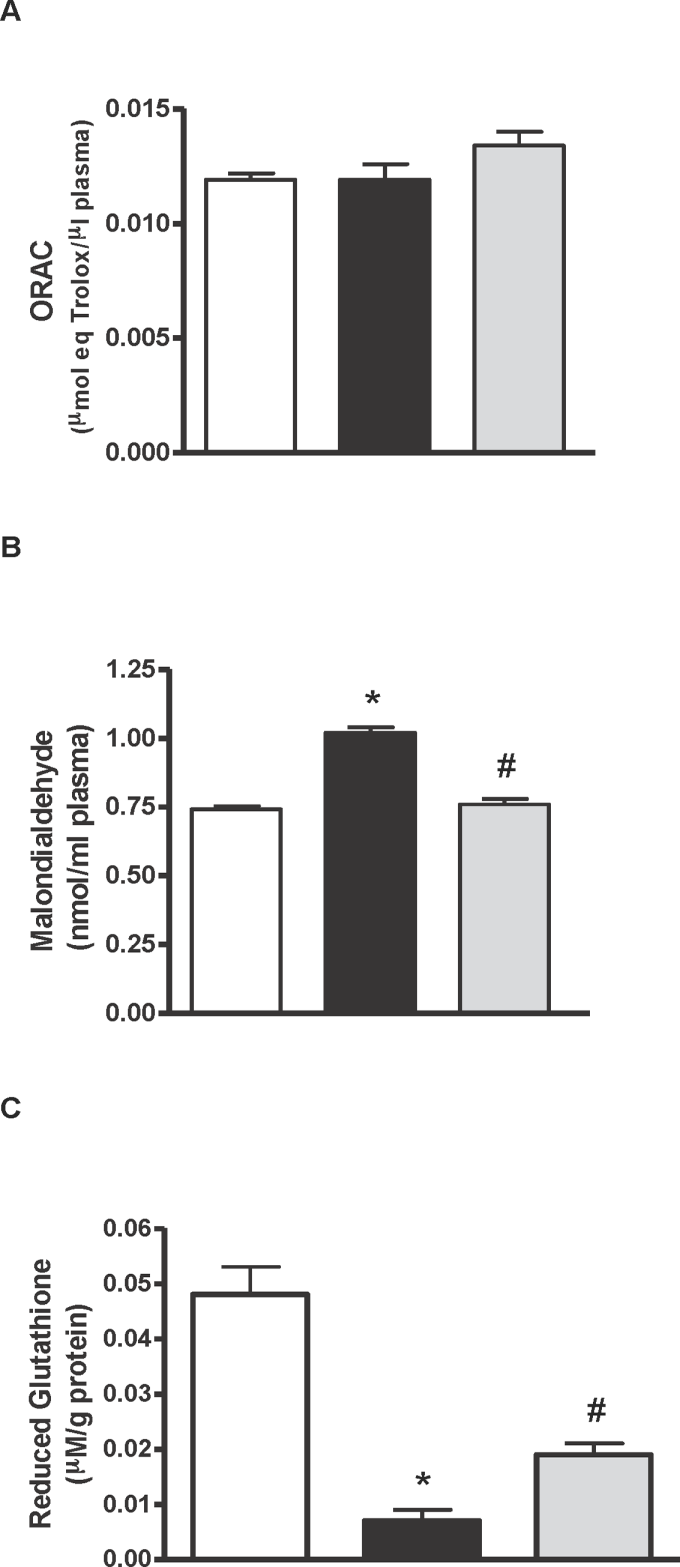
Antioxidant activity, MDA and reduced glutathione. Histograms of A) antioxidant activity (μmol eq Trolox/μl), B) malondialdehyde (nmol MDA/ml) in plasma and C) reduced glutathione (μM/g protein) in liver of lean Zucker rats (white), obese Zucker rats that received water (black) and obese Zucker rats that received 750 mg/kg/day of pepsin egg white hydrolysate (light grey) for 12 weeks. Values are means ± SEM (n = 10). * P<0.05 obese vs lean rats, #P<0.05 obese rats treated with pepsin egg white hydrolysate vs control obese rats.

Obese Zucker rats that took water presented elevated plasma TNF-α (Fig 5A) and adiponectin (Fig 5B) concentrations in comparison with the lean animals, and these parameters were significantly reduced in the obese rats that received the hydrolysate of egg white with pepsin. Regarding the antioxidant status of the animals, there were no differences in the radical scavenging capacity of plasma between lean and obese animals, although the obese group that took egg white hydrolysed with pepsin showed a positive trend in this parameter (Fig 6A). The plasma concentration of MDA, which is a lipid peroxidation index, was higher in the obese Zucker rats that took water than in the lean rats. The MDA concentration in plasma significantly decreased with the administration of egg white hydrolysed with pepsin (Fig 6B). Similarly, the intake of egg white hydrolysed with pepsin increased the levels of reduced glutathione in the liver of the obese animals (Fig 6C).

## Discussion

Obese Zucker rats, homozygous for the *fa* allele, present a mutation of the leptin receptor, which is the molecular basis for their characteristic phenotype [18]. In agreement with previous reports, in our study, the control obese Zucker rats developed hyperphagia during the first 14 weeks of life (Fig 1A) [19] and associated severe obesity (Fig 2) [20, 21]. By the 14^th^ week of life, the body fat percentage of obese Zucker rats reaches 40% [22], and this severe overweight probably restricts their mobility and access to food. These animals also exhibit a range of endocrine disorders, such as insulin resistance, glucose intolerance and hyperinsulinemia, which result in renal damage [23]. In fact, the increased diuresis observed in our work (Table 1) is probably a premature sign of diabetic nephropathy [24].

The observation that the proportion of FAD was higher in the obese rats as compared with the lean rats (Table 1), suggested that obesity in these animals could be associated with a more efficient fat digestion that could lead to fat accumulation. We observed an increased proportion of adipose tissue in the obese animals (Table 2, Fig 3), but also a substantial fat accumulation in non-adipose tissues, such as the liver, causing hepatomegaly (Table 2, Fig 4). In fact, increased lipogenesis and reduced fatty acid oxidation in the liver of obese Zucker rats determine the metabolism of triglycerides and the subsequent steatosis [25, 26]. In accordance with previous reports [27], in our study, the obese animals also presented dyslipidemia, as judged by the high plasma concentrations of cholesterol, triglycerides and free fatty acids, compared with their lean counterparts (Table 3).

In our study, the plasma levels of adiponectin were higher in the obese than in the lean Zucker rats (Fig 5B). Whereas it has been reported that obesity and insulin resistance are related with low plasma adiponectin concentrations in this animal model, and that restoration of plasma adiponectin levels are associated with a decrease in body weight gain and reduced hyperinsulinema and dyslipidemia [20, 21, 28], Vendrame et al. and Oana et al. [28, 29] also observed that obese Zucker rats of 13–17 weeks of age presented higher adiponectin levels than their lean counterparts. This situation of high circulating adiponectin could be related to a possible adiponectin resistance caused by a reduced expression of adiponectin receptors in certain tissues, such as the liver [30, 31] or the adipose tissue [32].

Obesity is associated with a state of chronic inflammation characterized by an abnormal production of proinflammatory mediators by the fat tissue, such as TNF-α [33], as shown in our work (Fig 5A). In fact, overexpression of TNF-α in obese Zucker rats induces activation of NADPH oxidase and production of ROS, leading to oxidative stress and endothelial dysfunction [34]. The radical scavenging activity of the plasma, as estimated by the ORAC test, was similar in the obese and lean Zucker rats, however, obese rats had elevated concentrations of plasma MDA, probably reflecting a higher level of lipid peroxidation (Fig 6A and 6B). On the other hand, reduced glutathione in the liver was ten times lower in the obese than in the lean Zucker rats (Fig 6C), indicating oxidative injury and, in general terms, a redox state typical of the extreme overweight condition of these animals.

The administration of egg white hydrolysed with pepsin and aminopeptidase led to a significantly reduced body weight in the obese Zucker rats during the first 4 weeks of the study (Fig 2). It is likely that this initial positive effect on body weight gain was further masked by the pronounced tendency of these animals to develop obesity. However, none of the hydrolysates reduced food intake by the obese rats (Fig 1A). Food proteins, and among them, egg proteins induce satiety and reduce energy intake [35]. In addition, protein hydrolysates may exhibit a most pronounced satiating effect by an enhanced triggering of the release of digestive hormones that modulate digestive motility, coordination of appetite and glucose homeostasis, although it has also been described that certain protein hydrolysates can decrease, in the short term, the rate of body weight gain independently of food consumption [36, 37].

Regarding fat accumulation in adipose and non-adipose tissues, the most promising results were obtained with the hydrolysate of egg white with pepsin. The weight of the epididymal adipose tissue was lower in the animals that were fed this hydrolysate than in the obese animals that drank just water (Table 2). On the other hand, the intake of the hydrolysate of egg white with pepsin significantly improved the hepatic steatosis typical of obese Zucker rats (Fig 4). Conversely, the hydrolysate of egg white with aminopeptidase was not effective in reducing fat accumulation or hepatic steatosis (Table 2 and Fig 3).

The hydrolysate of egg white with pepsin lowered the plasmatic concentration of FFAs and it also diminished the pro-inflammatory state, decreasing the plasma levels of TNF-α (Table 3 and Fig 5A). On the other hand, its normalizing effect on the plasma concentration of adiponectin (Fig 5B) suggests that it might have ameliorated adiponectin resistance, although expression of its receptors was not measured. Adiponectin effectively reduces fat accumulation by improving fatty acid oxidation and decreasing fatty acid synthesis [26]. Nevertheless, it should be mentioned that several studies reporting the beneficial effects of other products, such as antioxidants or fermentable fibers, on the metabolic alterations typical of obese Zucker rats refer that the reduction in FFAs and TNF-α is accompanied by an increased secretion of adiponectin [38–41]. This hydrolysate was able to reduce the oxidative stress that also characterizes the obese animals of this breed, decreasing the levels of lipid peroxidation products in serum and increasing the liver antioxidant load (Fig 6). Reductions of oxidative damage in the liver by potent antioxidant polyphenols have been directly related to their beneficial effects on fatty liver [26, 41].

The regulation of lipid metabolism is crucial in the bioactivity of certain food proteins, such as soy and fish proteins and their hydrolysates that prevent the development of fatty liver in Zucker rats [42, 43]. Fish protein hydrolysates influence hepatic fatty acid composition by stimulating the oxidation of fatty acids and the activity of antioxidant enzymes in the liver [44–46]. Administration of a lysozyme hydrolysate with alcalase did not modify body weight, nor improved the metabolic parameters of obese diabetic Zucker rats, but it exerted beneficial effects on renal damage and endothelial dysfunction, at least partially through a decreasing effect on oxidative stress [11].

In view that the consumption of the hydrolysate of egg white with pepsin did not affect body weight at the end of the study, its positive effects on fat accumulation and hepatic steatosis could be attributed to other metabolic properties. In fact, the different outcomes of the administration of both hydrolysates, produced with pepsin or aminopeptidase, pointed at their differential peptide composition as responsible for the activity [13]. The hydrolysate of egg white with pepsin contains peptides that possess *in vitro* and *in vivo* ACE-inhibitory activity [47, 48], as well as peptides with radical scavenging activity or able to inhibit low density lipoprotein oxidation, which ameliorate the blood lipid profile and oxidative status of SHR [7, 10]. It is likely that the hydrolysate of egg white with pepsin acted by enhancing the consumption of FFAs in the hepatocytes rather than promoting a compensatory increase in hepatic lipogenesis, as judged by the lack of a significant effect on circulating triglycerides and fat secretion (Tables 1 and 3). In addition, it could have contributed to a lower concentration of reactive oxygen species in the liver, reducing hepatic injury.

Overall, our results point at a beneficial effect of egg white hydrolysed with pepsin in fat accumulation, hepatic steatosis and dyslipidemia. Further investigation is needed to identify the mechanisms and pathways implicated in the effect produced by this hydrolysate. For this purpose its relation with insulin resistance in the obese Zucker rats is currently the focus of our investigation.
